# Serum and Vascular Stiffness Biomarkers Associated with the Severity of Degenerative Aortic Valve Stenosis and Cardiovascular Outcomes

**DOI:** 10.3390/jcdd9060193

**Published:** 2022-06-17

**Authors:** Jakub Baran, Łukasz Niewiara, Jakub Podolec, Mateusz Siedliński, Ewelina Józefczuk, Anna Bernacik, Rafał Badacz, Tadeusz Przewłocki, Piotr Pieniążek, Krzysztof Żmudka, Jacek Legutko, Anna Kabłak-Ziembicka

**Affiliations:** 1 Department of Interventional Cardiology, The John Paul II Hospital, Prądnicka 80, 31-202 Krakow, Poland; jakub_baran@yahoo.pl (J.B.); lniewiara@gmail.com (Ł.N.); jakub.podolec@uj.edu.pl (J.P.); abernacik@gmail.com (A.B.); rbadacz@gmail.com (R.B.); tadeuszprzewlocki@op.pl (T.P.); kardio@kki.krakow.pl (P.P.); zmudka@icloud.com (K.Ż.); jacek.legutko@uj.edu.pl (J.L.); 2 Department of Interventional Cardiology, Institute of Cardiology, Jagiellonian University Medical College, Św. Anny 12, 31-008 Krakow, Poland; 3 Department of Emergency Medicine, Faculty of Health Sciences, Jagiellonian University Medical College, Michałowskiego 12, 31-126 Krakow, Poland; 4 Department of Internal and Agricultural Medicine, Faculty of Medicine, Jagiellonian University Medical College, Skarbowa 1, 31-121 Krakow, Poland; mateusz.siedlinski@uj.edu.pl (M.S.); ewelina.jozefczuk@doctoral.uj.edu.pl (E.J.); 5 Department of Cardiac and Vascular Diseases, Institute of Cardiology, Jagiellonian University Medical College, Św. Anny 12, 31-008 Krakow, Poland; 6 Noninvasive Cardiovascular Laboratory, The John Paul II Hospital, Prądnicka 80, 31-202 Krakow, Poland

**Keywords:** degenerative aortic valve stenosis, serum biomarkers, surgical aortic valve replacement, transcatheter aortic valve replacement, vascular stiffness

## Abstract

Background: Although degenerative aortic valve stenosis (DAS) is the most prevalent growth-up congestive heart valve disease, still little known about relationships between DAS severity, vascular stiffness (VS), echocardiographic parameters, and serum biomarkers in patients undergoing transcatheter (TAVR) or surgical aortic valve replacement (SAVR). The objective of this study was to identify biomarkers associated with DAS severity, and those that are associated with cardiovascular death (CVD) and episodes of chronic heart failure (CHF) exacerbation. Methods: A total of 137 patients with initially moderate-to-severe DAS were prospectively evaluated for the relationship between DAS severity, baseline VS, and serum biomarkers (uPAR, GDF-15, Gal-3, IL-6Rα, ET-1, PCSK9, RANTES/CCL5, NT-proBNP, and hs-TnT), and were followed-up for 48 months. The prognostic significance of each variable for CVD and CHF risk was measured by hazard ratio of risk (HR), which was calculated by Cox’s proportional hazard model. Results: DAS severity showed correlations with IL-6Rα (r = 0.306, *p* < 0.001), uPAR (r = 0.184, *p* = 0.032), and NT-proBNP (r = −0.389, *p* < 0.001). Levels of ET-1 and Gal-3 were strongly correlated with VS parameters (r = 0.674, *p* < 0.001; r = 0.724, *p* < 0.001). Out of 137 patients, 20 were referred to TAVR, 88 to SAVR, and 29 to OMT. In TAVR patients, the highest levels of ET-1, Gal-3, and VS were found as compared to other patients. The highest incidence of CVD was observed in patients who underwent TAVR (35%), compared to SAVR (8%) and OMT (10.3%) (*p* = 0.004). In a multivariate analysis, ET-1 occurred predictive of CVD risk (HR 25.1, *p* = 0.047), while Gal-3 > 11.5 ng/mL increased the risk of CHF exacerbation episodes requiring hospital admission by 12%. Conclusions: Our study indicated that ET-1 and Gal-3 levels may be associated with the outcomes in patients with DAS.

## 1. Introduction

Degenerative aortic valve stenosis (DAS) is the most prevalent growth-up heart valve disease [1,2]. Late presentation of symptoms related to the development of severe DAS is inevitably associated with a higher complication rate and unfavorable outcomes resulting from the concomitant comorbidity [3,4].

Echocardiography remains the gold standard for diagnosis and evaluation of DAS severity, carrying interventional recommendations for the surgical (SAVR) or transcatheter aortic valve replacement (TAVR) [1]. However, echocardiographic parameters give little insight into DAS pathophysiology and have poor predictive value for the rate of progression from early- to late-stage disease and cardiovascular outcomes [5].

Herein, a contemporary approach to stratify cardiovascular risk and timing of intervention could be improved through the implementation of more complex work-ups. Further research might be focused on the evaluation of serum biomarkers associated with calcific DAS and imaging modalities, like magnetic resonance imaging, 3D printing, or vascular stiffness (VS) parameters [5,6,7,8,9,10].

This attitude seems reasonable, since pathologic mineralization of valve leaflets in calcific DAS involves complex relationships with both innate and adaptive immunity [11]. It was previously observed that the increased expression of tumor necrosis factor-alpha (TNF-α) and Interleukin 6 and its membrane receptor alpha (IL-6Rα) identified in the calcified aortic valve leaflets initiates an osteogenic process, and the mineralization of valve interstitial cells [12,13]. The latter induces over-production of leukotrienes, which may further amplify the inflammatory response in DAS [14]. More recent studies indicate a role of novel soluble biomarkers in the calcification process, including Galectin-3, fatty acids concentration on aortic leaflets, and non-coding RNAs involved in calcifications [15,16,17].

On the other hand, an inevitable DAS progression leads to myocardial damage and the left ventricle remodeling through the changes in the cellular architecture of the myocardium, hypertrophy, and eventually cardiomyocyte death. Few circulating biomarkers produced by cardiac tissue correlate with myocardial damage, including plasma B-type natriuretic peptide, growth/differentiation factor-15 (GDF-15), Endothelin 1 (ET-1), and cardiac troponin [18,19,20].

Although many research studies are running, still there is little known about relationships between DAS severity, VS, echocardiographic parameters, and serum biomarkers in patients referred to SAVR or TAVR.

Therefore, the objective of this study was to identify biomarkers associated with DAS severity, and those that are associated with cardiovascular death (CVD) and episodes of chronic heart failure (CHF) exacerbation.

## 2. Materials and Methods

### 2.1. Study Population

The study group comprised 137 consecutive patients with moderate-to-severe DAS (aortic valve area, AVA < 1.5 cm^2^) admitted to complex diagnostic work-ups before referral to SAVR or TAVR. Additional inclusion criteria were left ventricular ejection fraction (LVEF) ≥ 50% and negative history of stroke or transient ischemic attack (no neurological symptoms present or past, and/or ischemic lesions on brain CT scans confirming the cerebral ischemia as ensured by the consulting neurologist).

The exclusion criteria included: significant stenosis of any carotid or vertebral artery (exceeding 50% lumen reduction), persistent or chronic atrial fibrillation, concomitant mitral valve diseases, newly diagnosed or recent myocardial infarction (<3 months), hemodynamic instability, aortic dissection, and lack of informed consent.

On admission, all patients underwent evaluation for cardiovascular risk factors, echocardiographic study, and examination of carotid and vertebral arteries for the assessment of vascular stiffness parameters. The biochemical blood samples for serum biomarkers were taken during the hospital stay, as soon as informed consent was obtained. Serum biomarkers included GDF-15, ET-1, IL-6Rα, urokinase activator receptor (uPAR), galectin-3 (Gal-3), pro-protein convertase subtilisin/kexin 9 (PCSK9), chemokine ligand 5 (RANTES/CCL5), N-terminal prohormone of brain natriuretic peptide (NT-proBNP), and high-sensitivity troponin T (hs-TnT).

**Figure 1:**
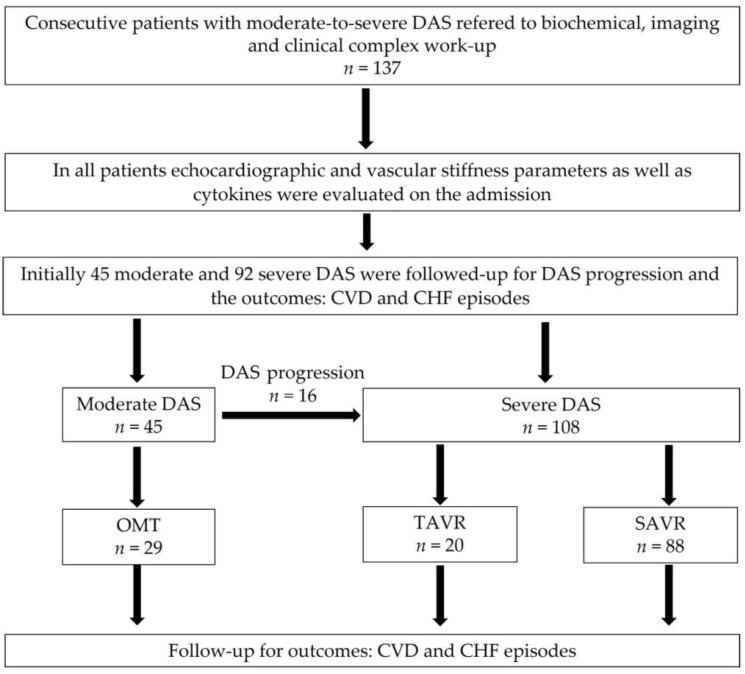
Study flowchart. Abbreviations: CHF, chronic heart failure; CVD, cardiovascular death; DAS, degenerative aortic stenosis; OMT, optimal medical treatment.

Based on the collected data, patients were referred to TAVR or SAVR according to the decision of the Heart Team consultation [21]. In the case of moderate DAS, they were referred to the observational group with a tight follow-up evaluation for symptom occurrence and progression to severe DAS (Figure 1).

Incidence of cardiovascular adverse events, including CVD and hospital re-admissions for exacerbation of CHF, were recorded prospectively during the 48-month follow-up period.

The study protocol was consistent with the requirements of the Helsinki Declaration and approved by the local Institutional Ethics Committee (KBET/118/B/2014 and KBET/1072.6120.148.2018). All patients signed the informed consent to participate in the study.

### 2.2. Cardiovascular Risk Factors and Echocardiographic Study

The prevalence of cardiovascular risk factors such as age, gender, hypertension, hyperlipidemia, smoking habit, and type 2 diabetes mellitus was evaluated in compliance with guidelines of the European Society of Cardiology [22,23].

All patients underwent a complete echocardiographic evaluation in compliance with guidelines of the European Association of Cardiovascular Imaging, focused on the left ventricular systolic and diastolic function, and aortic valve parameters [24]. LVEF was established according to the Simpson method from 4- and 2-chamber apical views. The diastolic function was established from the E and A mitral spectrum pattern and the medial and lateral mitral annulus velocity parameters with Tissue Doppler Imaging (TDI). The E/e’ ratio was defined as the proportion equation of E wave velocity to averaged medial and lateral e’ velocity on TDI. Echocardiographic parameters were measured by ultrasonographers blinded to the subject’s characteristics.

### 2.3. Vascular Stiffness Assessment

VS parameters, including Resistive and Pulsatile indices (RI, PI), were evaluated with a high-resolution B-Mode, color Doppler, and pulse Doppler ultrasonography of both carotid and vertebral arteries with an ultrasound machine (TOSHIBA APLIO 450) equipped with a linear-array 5–10 MHz transducer on a patient lying in the supine position with the head tilted slightly backward. Bilateral peak-systolic (PSV) and the end-diastolic (EDV) velocities were measured within the internal carotid artery and proximal V2 segment of the vertebral artery. RI and PI indices were evaluated according to mathematical formulae: Resistive Index (RI) = (PSV − EDV/PSV), and Pulsatile Index (PI) = PSV − EDV/((PSV + 2 × EDV)/3). Carotid and vertebral arterial flow parameters were measured by ultrasonographers blinded to the subject’s characteristics.

### 2.4. Cytokines Assessment

During index hospitalization, fasting blood was collected from an antecubital vein and placed in a tube. Within 30 min of blood collection, plasma was centrifuged for 15 min at 1600× *g* at 4 °C. Collected serum aliquots were immediately stored at ≤−70 °C until analysis. Concentration of proteins in serum was measured using commercially available ELISA kits according to manufacturer instructions (no. DRN00B (RANTES/CCL5), DPC900 (PCSK9), DET100 (Endothelin 1), and DR600 (IL6R alpha) from R&D Systems and BMS279-4 (Galectin-3), EHGDF15 (GDF-15), and EHPLAUR (uPAR) from ThermoFisher Scientific). Final absorbance readings were obtained using a Synergy H4 Hybrid Multi-Mode Microplate Reader (Thermo Fisher Scientific, Waltham, MA, USA).

### 2.5. Follow-Up Period

During an observation period of 48 months, aortic valve progression and the incidence of combined end-point defined as CVD and/or exacerbation episodes of CHF were recorded. CVD was defined as fatal (ischemic stroke, myocardial infarction, acute heart failure episode) or other CVD (i.e., any sudden or unexpected death unless proven as non-cardiovascular on autopsy). CHF episodes were defined as hospitalization for newly diagnosed exacerbated CHF requiring the administration of intravenous diuretics and/or vasoactive drugs (dopamine, dobutamine, epinephrine, or norepinephrine).

The final follow-up (FU) visit was performed as telephone visit with the patient or mandated family member. For all patients, data regarding patient vital status were obtained from the national health registry at the closing database.

### 2.6. Statistical Analysis

Data are presented as mean ± standard deviation or median [interquartile range] for continuous variables and as proportions for categorical variables. Normal distribution of the studied variables was determined by the Shapiro-Wilk test. Differences between mean values were verified using the Student’s *t*-test and Analysis of Variance (ANOVA) test, while frequencies were compared using the Chi-squared test for independence, as appropriate.

A univariate Cox proportional hazards analysis was used to determine risk factors for CVD and CHF exacerbation episodes, including traditional cardiovascular risk factors, echocardiographic and VS parameters, and serum biomarkers. For those parameters reaching a *p*-value of less than 0.1, a multivariate Cox proportional hazards analysis was performed with the forward-backward stepwise selection method. For continuous variables, we tried receiver operating characteristic (ROC)-determined biomarker cut-points, being defined as the threshold value of the continuous covariate distribution, which best separates low- and high-risk patients with respect to the outcome. For the ‘optimal’ cut-off value, the area under the curve (AUC), 95% Confidence Intervals (95% CI), as well as a sensitivity and specificity were calculated.

Statistical analyses were performed with Statistica version 13.3 software (TIBCO Software, Palo Alto, CA, USA) and with R Studio 3.6.3 [25].

## 3. Results

### 3.1. Baseline Patients’ Characteristics

Out of 137 consecutive patients admitted with the intention of TAVR or SAVR, as a result of a complex diagnostic work-up, severe DAS (AVA < 1.0 cm^2^) was confirmed in 92 patients, while in 45 patients, DAS was classified as moderate (AVA between 1.01 and 1.5 cm^2^).

Patients with severe vs moderate DAS did not differ with regard to cardiovascular risk factors distribution, except from hyperlipidemia (*p* = 0.04), and lower extremities arterial disease (LEAD) (*p* = 0.034) (Table 1). There were significant differences with respect to echocardiographic parameters, such as aortic valve gradients, left ventricle wall thickness, and LVEF, as well as symptoms in a higher New York Heart Association Functional Class (NYHA) between moderate and severe DAS (Table 1). There was a trend showing a difference in baseline VS parameters and hs-CRP level. Significant differences in median levels of ET-1 (*p* = 0.001), IL-6R ά (*p* < 0.001), NT-pro-BNP (*p* = 0.004), and uPAR (*p* = 0.031) were observed between patients with moderate vs. severe DAS (Table 1).

### 3.2. Associations between DAS Severity and DAS Progression with Serum Biomarkers, Echocardiographic and VS Parameters

DAS severity showed weak, yet significant positive correlations with IL-6Rα (r = 0.306, *p* < 0.001), uPAR (r = 0.184, *p* = 0.032), LVEF (r = 0.223, *p* = 0.009), and VS (r = 0.211, *p* = 0.025), while showing a negative correlation with NT-proBNP (r = −0.389, *p* < 0.001). We found good correlation between Gal-3 levels and VS parameters (r = 0.674, *p* < 0.001), as well as between ET-1 and VS (r = 0.724, *p* < 0.001).

In patients with initially moderate DAS, DAS progression to severe aortic valve stenosis showed association with RANTES/CCL5 (*p* = 0.01), but not with other serum or VS biomarkers (Table 2). Among echocardiographic parameters, a lower left ventricular end-diastolic diameter (LVEDD), greater left ventricle posterior wall thickness, and lower e’ lateral velocity were observed in patients who had a progression to severe DAS during the follow-up period (Table 2).

### 3.3. Comparison of Studied Parameters between SAVR, TAVR, and OMT Groups

Eventually, out of 137 patients, 88 and 20 underwent SAVR and TAVR, respectively. A total of 29 patients with non-severe DAS were referred to optimal medical treatment (OMT) and the observational group.

Not surprisingly, TAVR patients were significantly older (*p* < 0.001) and presented symptoms in a higher NYHA class (Table 3). Additionally, paroxysmal atrial fibrillation was more prevalent in the TAVR group. In addition, levels of serum creatinine, ET-1, NT-pro-BNP, and Gal-3 were significantly higher in the TAVR group as compared to the other groups of patients, while eGFR was the lowest in the TAVR group (Table 3).

### 3.4. Cardiovascular Outcomes

During a follow-up period of 48 months, CVD was recorded in 17 (12.4%) patients, while an exacerbation of CHF requiring hospitalization was recorded in 23 (16.8%) patients.

After the intervention, the highest incidence rate of CVD was observed in patients who underwent TAVR, as compared to the SAVR and OMT groups (35% vs. 8% and 10.3%, *p* = 0.004, respectively). The incidence of CHF did not differ significantly between study groups (25% vs. 17.1% and 10.3%, *p* = 0.400, respectively). Additionally, a comparison between the TAVR and SAVR groups showed a statistical difference in CVD rates (*p* = 0.003), while it showed no difference in the prevalence of CHF episodes (*p* = 0.683).

In a univariate Cox proportional hazard ratio analysis, age, LEAD, NYHA class III, ET-1, IL-6Rα, NT-proBNP, hs-TnT, serum creatinine, and eGFR showed associations with risk of CVD (Table 4). The ROC-determined optimal cut-point for NT-pro-BNP was >500 pg/mL (AUC 0.684 (95% CI 0.554–0.813), *p* = 0.005), with a sensitivity of 57% and a specificity of 43%.

In the multivariate analysis, age, LEAD, NYHA class III, ET-1, IL-6Rα, hs-TnT, serum creatinine, and eGFR retained their associations with CVD risk (Table 4).

With regard to episodes of CHF exacerbation, in a univariate Cox analysis, biomarkers associated with CHF risk were female gender, increased VS, left atrium area, diastolic dysfunction (E/e’), Gal-3, and eGFR. The ROC-determined optimal cut-point for Gal-3 was 11.5 ng/mL (AUC 0.711 (95% CI 0.602–0.819), *p* < 0.001), with a sensitivity of 87% and a specificity of 51%. For VS, the RI ≥ 0.7 and the PI ≥ 1.3 were established as optimal cut-offs (AUC 0.698 (95% CI 0.582–0.813), *p* < 0.001), with a sensitivity of 71% and a specificity of 63%.

In a multimarker score developed using a Cox proportional hazards model for CHF exacerbation episode incidences and ROC-determined biomarker cut-points, only Gal-3 higher than 11.5 ng/mL was associated with a 12% risk increase of hospital admissions for exacerbated CHF (95% CI 1.01–1.25) (Table 4).

## 4. Discussion

Our working hypothesis was that specific serum biomarkers and VS parameters may be of help in the decision-making processes in DAS management.

The first aspect of our study concerned the relationship between DAS severity and analyzed serum and VS biomarkers. We have observed weak, yet significant associations between DAS severity and levels of IL-6Rα and uPAR. We also found a higher median level of ET-1 in patients with severe DAS, compared to lower DAS severity.

Recent studies showed that the calcification extent in aortic leaflets was associated with the increased secretion of soluble uPAR, a biomarker predictive of cardiovascular outcomes after the intervention on the valve [26,27].

The pro-inflammatory molecule involved in vascular osteogenesis is Gal-3, which was found to be an important biomarker of DAS calcification severity [16]. Conflicting data were reported by Arangalage et al. who, in a group of 558 patients with DAS, did not find Gal-3 to provide prognostic information on functional status or DAS severity [28].

Although we did not observe a direct relationship between DAS severity and Gal-3 concentration, patients referred to TAVR had significantly higher median levels of Gal-3 as compared to other patients. Similarly, in a study by Bobrowska et al., the median Gal-3 levels were higher in patients with critically-ill aortic valve stenosis referred to balloon aortic valvuloplasty (BAV), with further prospective TAVR [29]. In the mentioned study, there was a trend to significance for all-cause mortality in patients with Gal-3 levels exceeding 17.8 ng/mL irrespective of the type of treatment employed (the log rank *p* = 0.09) [30].

Moreover, in our present study, Gal-3 was the only independent risk factor associated with episodes of CHF exacerbations during the follow-up period. In a multimarker score developed using a Cox proportional hazards model for CHF exacerbation episode incidence, Gal-3 levels higher than 11.5 ng/mL were associated with a 12% increase in the risk of hospital readmissions for exacerbated CHF. The possible explanation of Gal-3’s role in the worsening of CHF links Gal-3 with renal and cardiac fibrosis resulting from the cardiac and vascular remodeling, and aldosterone increase.

Importantly, in experimental animal models, inhibition of Gal-3 blocked aortic valve calcification, cardiac and vascular fibrosis and inflammation [30,31]. These data suggest a key role for Gal-3 in cardiorenal remodeling and dysfunction induced by aldosterone. Moreover, targeting Gal-3 may be an upstream therapeutic option for the treatment of aortic valve and cardiovascular remodeling that accompanies the progression of DAS [30,31,32].

On the other hand, there are data on the protective role of some biomarkers, such as the secretion of the anti-inflammatory cytokine Interleukine 10 stimulated by the expression of toll-like receptor 7 driving M2 macrophages subset activation [33]. Additionally, carotid intima-media thickness can rule out those patients with a calcification process limited to aortic leaflet valves from those with DAS and significant obstructive lesions in coronary arteries [34].

The other serum biomarker involved in the endothelial to mesenchymal cells transformation, the descending regulation of vasodilation and vasoconstriction, and vascular inflammation is ET-1 [35]. It was evidenced previously that ET-1 could be identified as an independent predictor of the presence of stage 3 and/or 4 of DAS severity, like pulmonary vasculature or tricuspid valve damage (Stage 3), or right ventricular damage (Stage 4) [36,37].

As we have evidenced in the present study, the levels of ET-1 and Gal-3 were strongly correlated with VS parameters, and more importantly the highest levels of ET-1, Gal-3, and VS were found in patients referred to TAVR. Consistently, the highest incidence rate of CVD was observed in patients who underwent TAVR (35%), as compared to the SAVR (8%) and OMT (10.3%) groups (*p* = 0.004). In line, we have found that ET-1 levels were strongly and independently predictive of CVD risk (HR 25.1, *p* = 0.047) in a multivariate Cox proportional hazard analysis.

We have also found several positive correlations between DAS severity and LVEF (r = 0.223, *p* = 0.009), while we found a negative one with NT-proBNP (r = −0.389, *p* < 0.001). It is reasonable, as progressing DAS leads eventually to left ventricle damage, NYHA symptom occurrence, and the exacerbation of CHF, resulting in the increased release of NT-proBNP.

In our present study, hs-TnT, but not NT-proBNP, occurred as an independent risk factor of CVD in patients with DAS during the follow-up period. Cardiac troponin was validated as one of several variables associated with the myocardium fibrosis, a risk factor of cardiovascular events such as CVD, and heart failure episodes with a high sensitivity and specificity [38,39]. Additionally, the other independent risk factors associated with CVD incidence in this study such as age, LEAD, and renal function parameters are commonly recognized risk factors in patients with a broad spectrum of cardiovascular diseases, including atherosclerosis, valve disease, and cardiomyopathies [40,41,42].

In conclusion, our study indicated that ET-1 and Gal-3 levels may be associated with the outcomes in patients with DAS.

However, our present study has several limitations such as single-center design and the limited number of study participants. Additionally, the other biomarkers that have a role in phosphoro-calcium bone metabolism such as osteoprotegerin and osteopontin may be worthy of investigation [43,44]. Additionally, further studies, perhaps multicenter or longitudinal, would be needed to confirm our findings.

## Figures and Tables

**Table 1:** Baseline study groups’ characteristics.

	All Study Participants N = 137	Moderate DAS N = 45	Severe DAS N = 92	*p*-Value *
Demographic data				
Age, years, ± SD	69.5 ± 8.5	69.2 ± 8.37	69.7 ± 8.66	0.727
BMI, ± SD	29.6 ± 5.60	30.9 ± 5.28	28.9 ± 5.69	0.062
Female, n (%)	67 (48.9%)	25 (55.6%)	42 (45.7%)	0.364
Hypertension, n (%)	130 (94.9%)	44 (97.8%)	44 (97.8%)	0.426
Type 2 Diabetes, n (%)	40 (29.2%)	13 (28.9%)	27 (29.3%)	1
Hyperlipidemia, n (%)	132 (96.4%)	41 (91.1%)	91 (98.9%)	0.04
Paroxysmal atrial fibrillation, n (%)	21 (15.4%)	7 (15.6%)	14 (15.4%)	1
Smoking history, n (%)	36 (26.3%)	12 (26.7%)	24 (26.1%)	1
Previous MI, n (%)	11 (8.03%)	2 (4.44%)	9 (9.78%)	0.339
COPD, n (%)	11 (8.03%)	5 (11.1%)	6 (6.52%)	0.504
CAD, n (%) **	45 (32.8%)	17 (37.8%)	28 (30.4%)	0.545
Previous PCI, n (%)	28 (20.4%)	13 (28.9%)	15 (16.3%)	0.136
Previous CABG, n (%)	2 (1.46%)	1 (2.22%)	1 (1.09%)	0.551
LEAD, n (%)	27 (19.7%)	14 (31.1%)	13 (14.1%)	0.034
Anemia ***, n (%)	26 (19%)	6 (13.3%)	20 (21.7%)	0.239
Clinical symptom				
NYHA III vs. I + II, n (%)	29 (21.2%)	1 (2.22%)	28 (30.4%)	<0.001
Echocardiographic data				
Aortic valve area (cm^2^) ± SD	0.94 ± 0.31	1.28 ± 0.24	0.77 ± 0.17	<0.001
Peak aortic velocity (m/s) ± SD	4.29 ± 0.86	3.46 ± 0.57	4.70 ± 0.67	<0.001
Mean aortic gradient (mmHg) ± SD	47.6 ±19.8	28.9 ± 9.91	56.7 ± 16.9	<0.001
LVEF (%) ± SD	62.8 ± 7.33	64.7 ± 7.37	61.9 ± 7.17	0.038
LVEDD (mm) ± SD	47.4 ± 5.12	47.7 ± 4.87	47.2 ± 5.27	0.598
IVS thickness in diastole (mm) ± SD	13.5 ± 2.18	12.7 ± 1.72	14.0 ± 2.27	<0.001
PW thickness in diastole (mm) ± SD	12.0 ± 1.73	11.6 ± 1.48	12.2 ± 1.81	0.04
Left atrium (cm^2^) ± SD	24.4 ± 4.65	24.4 ± 5.18	24.5 ± 4.37	0.967
e’ medial velocity (cm/s) ± SD	6.71 ± 1.85	7.67 ± 1.67	6.21 ± 1.76	0.024
e’ lateral velocity (cm/s) ± SD	7.49 ± 2.59	7.94 ± 1.98	7.27 ± 2.86	0.475
E/e’, 1 ± SD	11.8 ± 2.81	12.2 ± 3.10	11.6 ± 2.65	0.319
Tricuspid regurgitant velocity (m/s) ± SD	2.63 ± 0.43	2.61 ± 0.42	2.65 ± 0.44	0.696
Vascular stiffness parameters				
Resistive Index, median [Q1; Q3]	0.68 [0.63; 0.72]	0.70 [0.64; 0.72]	0.67 [0.61; 0.71]	0.09
Pulsatile Index, median [Q1; Q3]	1.24 [1.08; 1.37]	1.31 [1.13; 1.39]	1.24 [1.05; 1.37]	0.087
Biochemical parameters				
RANTES/CCL5 (ng/mL), median [Q1; Q3]	29.9 [21.8; 40.9]	28.5 [21.8; 36.4]	30.5 [21.8; 42.2]	0.272
ET-1 (pg/mL), median [Q1; Q3]	1.67 [1.42; 2.09]	1.56 [1.34; 1.88]	1.86 [1.44; 2.11]	0.001
Gal-3 (ng/mL), median [Q1; Q3]	11.9 [9.55; 15.4]	10.5 [8.68; 14.0]	12.3 [10.3;15.5]	0.169
GDF-15 (ng/mL), median [Q1; Q3]	0.98 [0.62; 1.45]	1.05 [0.62; 1.50]	0.87 [0.62; 1.27]	0.512
hsTnT (pg/mL), median [Q1; Q3]	14 [11; 22]	15 [10; 24]	13 [11; 22]	0.779
IL-6R ά (ng/mL), median [Q1; Q3]	46.8 [38.8; 58.9]	51.9 [44.4; 68.1]	43.1 [36.1; 54.3]	<0.001
PCSK9 (ng/mL), median [Q1; Q3]	308 [243; 352]	326 [244; 377]	286 [243; 333]	0.122
NT-pro-BNP (pg/mL), median [Q1; Q3]	487 [188; 823]	325 [143; 681]	571 [234; 1098]	0.004
uPAR (ng/mL), median [Q1; Q3]	1.44 [1.19; 1.86]	1.61 [1.28; 2.21]	1.42 [1.14; 1.73]	0.031
Creatinine (µmol/L), median [Q1; Q3]	79.0 [69.0; 95.0]	78.0 [66.0; 95.0]	80.5 [70.0; 95.2]	0.403
eGFR (mL/min/1.73 m^2^), median [Q1; Q3]	76.0 [64.0; 88.0]	79.0 [65.0; 87.0]	74.5 [63.0; 89.0]	0.67
Hemoglobin (mg/dL), median [Q1; Q3]	13.8 [12.6; 15.1]	13.7 [12.6; 14.9]	13.9 [12.7; 15.1]	0.971
hs-CRP (mg/L), median [Q1; Q3]	1.88 [0.98; 3.29]	2.36 [1.17; 5.06]	1.62 [0.93; 3.05]	0.052

* P-level provided between groups with moderate vs severe DAS; ** Coronary artery disease was defined as lesions exceeding 50% lumen reduction in at least one major coronary artery. *** Anemia was defined as Hemoglobin < 13 mg/dL in males and <12 mg/dL in females. Abbreviations: BMI, body mass index; CABG, coronary artery bypass graft; CAD, coronary artery disease; COPD, chronic obstructive pulmonary disease; DAS, degenerative aortic valve stenosis; eGFR, estimated glomerular filtration rate; ET-1, endothelin-1; Gal-3, galectin 3; GDF-15, growth/differentiation factor 15; hs-CRP, high sensitive C-Reactive Protein; hsTnT, high sensitive troponin T; IL-6R ά, interleukin 6 receptor alpha; IVS—intraventricular septum; LEAD, lower extremities artery disease; LVEDD, left ventricular end-diastolic diameter; LVEF, left ventricle ejection fraction; MI, myocardial infarction; NT-pro-BNP, N-terminal pro-B-type natriuretic peptide; NYHA, New York Heart Association Functional Classification; PCI, percutaneous coronary intervention; PCSK9, proprotein convertase subtilisin/kexin 9; PW—posterior wall; RANTES/CCL5, chemokine ligand 5; SD, standard deviation; uPAR, urokinase-type plasminogen activator receptor.

**Table 2:** Biomarkers associated with progression to severe DAS.

Biomarkers	Progression	Non-Progression	
	*n* = 16	*n* = 29	
Serum biomarkers			
RANTES/CCL5 (ng/mL), median [Q1; Q3]	32.2 [26.4; 40.8]	26.7 [17.0; 36.6]	0.006
ET-1 (pg/mL), median [Q1; Q3]	1.55 [1.25; 2.09]	1.56 [1.31; 1.87]	0.657
Gal-3 (ng/mL), median [Q1; Q3]	13.1 [9.6; 17.4]	10.0 [8.6; 13.5]	0.897
GDF-15 (ng/mL), median [Q1; Q3]	0.94 [0.64; 1.47]	1.04 [0.52; 1.53]	0.849
hsTnT (pg/mL), median [Q1; Q3]	15 [12; 17.5]	14 [10; 28]	0.841
IL-6R ά (ng/mL), median [Q1; Q3]	68.5 [49.8; 82.1]	50.2 [42.2; 57.1]	0.726
PCSK9 (ng/mL), median [Q1; Q3]	339.8 [275.9; 418.2]	314.3 [230.9; 371.3]	0.682
NT-pro-BNP (pg/mL), median [Q1; Q3]	158.0 [99; 407]	421.5 [147; 757]	0.177
uPAR (ng/mL), median [Q1; Q3]	1.63 [1.30; 2.21]	1.54 [1.18; 2.36]	0.897
LDL cholesterol (mmol/L), median [Q1; Q3]	2.96 [2.67; 3.96]	2.57 [2.23; 3.40]	0.465
Serum creatinine (µmol/L), median [Q1; Q3]	85 [70.2; 95.0]	72 [64.5; 99]	0.234
eGFR (mL/min/1.73 m^2^), median [Q1; Q3]	78 [57; 85]	80 [64; 88]	0.308
Hemoglobin (mg/dL), median [Q1; Q3]	13.7 [13.3; 14.4]	13.4 [12.4; 14.6]	0.976
hs-CRP (mg/L), median [Q1; Q3]	1.85 [0.95; 4.66]	2.87 [1.11; 5.62]	0.742
Vascular stiffness biomarkers			
PI, median [Q1; Q3]	1.33 [1.11; 1.44]	1.29 [1.20; 1.39]	0.968
RI, median [Q1; Q3]	0.70 [0.65; 0.74]	0.69 [0.66; 0.72]	0.936
Echocardiographic data			
LVEF (%), median [Q1; Q3]	65.5 [62.8; 72.3]	65 [61; 67.9]	0.258
LVEDD (mm), median [Q1; Q3]	46 [43.4; 49]	49 [46; 52]	0.048
IVS thickness in diastole (mm), median [Q1; Q3]	13 [12; 15]	12 [11; 13]	0.105
PW thickness in diastole (mm), median [Q1; Q3]	12 [11; 13]	11 [10; 12]	0.054
Left atrium area (cm^2^), median [Q1; Q3]	23 [21; 26]	24 [22; 27]	0.352
e’ medial velocity, median [Q1; Q3]	7 [6.0; 9.0]	8 [5.8; 9.3]	0.89
e’ lateral velocity, median [Q1; Q3]	5.8 [5.0; 7.6]	9.0 [6.8; 10.3]	0.028
E/e’, median [Q1; Q3]	14 [9.0; 15.8]	9.7 [8.0; 12.5]	0.509

**Table 3:** Detailed comparison of patients according to final referral to observational group (OMT), SAVR, and TAVR.

	OMT	SAVR	TAVR	*p*-Value
	N = 29	N = 88	N = 20	
Demographic data				
Age, years, ± SD	69.8 ± 8.36	67.1 ± 7.46	79.7 ± 5.46	<0.001
BMI, ± SD	30.7 ± 5.49	29.3 ± 5.84	29.5 ± 4.64	0.516
Female, n (%)	16 (53.3%)	39 (44.3%)	12 (63.2%)	0.284
Hypertension, n (%)	28 (96.6%)	82 (93.2%)	20 (100%)	0.626
Type 2 Diabetes, n (%)	8 (27.6%)	27 (30.7%)	5 (25.0%)	0.86
Hyperlipidemia, n (%)	26 (89.7%)	86 (97.7%)	20 (100%)	0.104
Paroxysmal atrial fibrillation, n (%)	6 (20.7%)	7 (8.05%)	8 (40.0%)	0.001
Smoking history, n (%)	7 (24.1%)	22 (25.0%)	7 (35.0%)	0.629
Previous MI, n (%)	1 (3.45%)	8 (9.09%)	2 (10.0%)	0.65
COPD, n (%)	4 (13.8%)	5 (5.68%)	2 (10.0%)	0.297
CAD, n (%)	7 (24.1%)	14 (15.9%)	7 (35.0%)	0.145
Previous PCI, n (%)	1 (3.45%)	0 (0.00%)	1 (5.00%)	0.126
Previous CABG, n (%)	8 (27.6%)	14 (15.9%)	5 (25.0%)	0.289
LEAD, n (%)				
Clinical symptom	0 (0.00%)	19 (21.6%)	10 (50.0%)	<0.001
NYHA III vs. I + II, n (%)				
Echocardiographic data				
Aortic valve area (cm^2^) ± SD	1.36 ± 0.24	0.83 ± 0.22	0.81 ± 0.16	<0.001
Peak aortic velocity (m/s), 1 ± SD	3.21 ± 0.45	4.62 ± 0.70	4.42 ± 0.71	<0.001
Mean aortic gradient (mmHg) ± SD	24.9 ± 7.83	54.7 ± 17.5	49.0 ± 17.3	<0.001
LVEF (%), 1 ± SD	63.7 ± 7.59	63.0 ± 7.60	61.0 ± 5.44	0.44
Left ventricular end-diastolic diameter (mm) ± SD	48.7 ± 5.02	47.0 ± 5.15	46.9 ± 5.11	0.288
IVS thickness in diastole (mm), 1 ± SD	12.3 ± 1.72	13.9 ± 2.15	13.9 ± 2.39	0.003
PW thickness in diastole (mm), 1 ± SD	11.2 ± 1.50	12.2 ± 1.82	12.2 ± 1.31	0.03
Left atrium (cm^2^), 1 ± SD	24.2 ± 3.92	24.3 ± 3.99	23.6 ± 3.55	0.673
e’ medial, 1 ± SD	7.67 ± 1.86	6.59 ± 2.03	6.20 ± 0.40	0.349
e’ lateral, 1 ± SD	8.67 ± 1.86	6.89 ± 2.68	8.10 ± 2.84	0.307
E/e’, 1 ± SD	11.7 ± 3.01	11.8 ± 2.71	11.9 ± 3.07	0.985
Tricuspid regurgitant velocity (m/s), 1 ± SD	2.56 ± 0.41	2.64 ± 0.45	2.73 ± 0.35	0.584
Vascular stiffness parameters				
Resistive Index, median [Q1; Q3]	0.69 [0.65; 0.72]	0.67 [0.61; 0.71]	0.74 [0.68; 0.77]	0.018
Pulsatile Index, median [Q1; Q3]	1.29 [1.16; 1.39]	1.22 [1.06; 1.36]	1.46 [1.24; 1.59]	0.02
Biochemical parameters				
RANTES/CCL5 (ng/mL), median [Q1; Q3]	26.7 [17.6; 36.3]	29.9 [22.9; 40.7]	32.8 [17.4; 45.1]	0.591
ET-1 (pg/mL), median [Q1; Q3]	1.56 [1.44; 1.78]	1.81 [1.59; 1.81]	1.81 [1.81; 1.84]	0.008
Gal-3 (ng/mL), median [Q1; Q3]	9.96 [8.7; 13.0]	11.8 [9.6; 15.5]	14.9 [11.7; 16.7]	0.04
GDF-15 (ng/mL), median [Q1; Q3]	1.04 [0.53; 1.51]	0.94 [0.62; 1.46]	1.13 [0.84; 1.27]	0.878
hsTnT (pg/mL), median [Q1; Q3]	14 [9.5; 22.0]	13 [10.7; 20.0]	17.5 [12.5; 33.2]	0.085
IL-6R ά (ng/mL), median [Q1; Q3]	50.2 [42.3; 56.5]	46.6 [38.8; 61.6]	39.9 [31.6; 53.2]	0.161
PCSK9 (ng/mL), median [Q1; Q3]	314 [232; 343]	301 [253; 354]	319 [296; 324]	0.931
NT-pro-BNP (pg/mL), median [Q1; Q3]	422 [147; 757]	392 [188; 784]	998 [540; 1698]	0.011
uPAR (ng/mL), median [Q1; Q3]	1.54 [1.19; 2.32]	1.42 [1.15; 1.73]	1.56 [1.23; 2.21]	0.329
Creatinine (µmol/L), median [Q1; Q3]	72.0 [65.0; 98.0]	79.0 [69.0; 92.2]	88.5 [78.5; 107]	0.026
eGFR (mL/min/1.73 m^2^), median [Q1; Q3]	80.0 [65.0; 88.0]	77.0 [67.0; 89.0]	56.5 [43.5; 69.8]	0.001
Hemoglobin (mg/dL), median [Q1; Q3]	13.9 [12.6; 15.6]	13.9 [13.0; 15.1]	12.9 [11.9; 13.7]	0.207
Hs-CRP (mg/L), median [Q1; Q3]	2.87 [1.27; 5.12]	1.90 [1.06; 3.08]	0.80 [0.55; 2.68]	0.07

**Table 4:** The univariate and multivariate Cox proportional hazard analysis of biomarkers associated with cardiovascular death and chronic heart failure exacerbation episodes.

	Cardiovascular Death		CHF	
	Univariate Cox	Multivariate Cox	Univariate Cox	Multivariate Cox
	HR (95% CI), *p*-Value	HR (95% CI), *p*-Value	HR (95% CI), *p*-Value	HR (95% CI), *p*-Value
Demographic data				
Age	1.06 (1.0–1.13), 0.039	0.71 (0.54–0.90), 0.014	1.04 (0.98–1.09), 0.122	-
BMI	0.99 (0.90–1.10), 0.958	-	1.04 (0.96–1.12), 0.297	-
Female gender	0.91 (0.35–2.36), 0.846	-	3.20 (1.26–8.14), 0.014	1.69 (0.29–9.82), 0.556
Hypertension	0.76 (0.10–5.82), 0.800	-	N/A	-
Diabetes	1.01 (0.35–2.86), 0.987	-	1.13 (0.46–2.76), 0.782	-
Paroxysmal atrial fibrillation	2.25 (0.72–7.01), 0.161	-	3.66 (1.40–9.57), 0.008	0.95 (0.12–7.01), 0.957
Smoking history	1.58 (0.60–4.17), 0.352	-	0.59 (0.22–1.59), 0.299	-
Previous MI	0.78 (0.10–5.92), 0.812	-	1.39 (0.32–6.03), 0.659	-
CAD	1.19 (0.44–3.23), 0.729	-	1.45 (0.62–3.37), 0.380	-
LEAD	3.54 (1.36–9.23), 0.009	109 (4.06–2976), 0.005	0.75 (0.27–2.05), 0.581	-
Clinical symptoms:	2.88 (90.98–8.51), 0.055	0.05 (0.10–0.50), 0.023	1.23 (0.35–4.32), 0.743	-
NYHA class III vs. I + II				
Echocardiographic data				
Aortic valve area	1.17 (0.25–5.40), 0.840	-	1.03 (0.27-.396), 0.958	-
LVEF	1.00 (0.94–1.07), 0.890	-	1.02 (0.96–1.09), 0.474	
LVEDD	0.94 (0.84–1.04), 0.252	-	1.00 (0.92–1.09), 0.906	-
IVS thickness in diastole	1.19 (0.97–1.46), 0.080	1.00 (0.37–2.90), 0.928	1.03 90.86–1.25), 0.675	-
PW thickness in diastole	1.02 (0.78–1.35), 0.854	-	1.12 (0.91–1.39), 0.273	-
Left atrium area	1.04 (0.94–1.15), 0.369	-	1.09 (1.01–1.19), 0.039	1.05 (0.96–1.14), 0.221
e’ medial	0.69 (0.32–1.53), 0.368	-	1.04 (0.71–1.52), 0.825	-
e’ lateral	0.96 (0.58–1.59), 0.886	-	0.86 (0.58–1.27), 0.443	-
E/e’	0.89 (0.70–1.15), 0.394	-	1.22 (1.04–1.42), 0.014	1.07 (0.86–1.32), 0.545
**Vascular stiffness parameters**				
Increased VS (RI ≥ 0.7 and PI ≥ 1.3)	1.72 (0.61–4.85), 0.304	-	2.80 (1.13–6.94), 0.026	2.25 (0.85–5.98), 0.110
**Biochemical parameters**				
RANTES/CCL5	0.98 (0.95–1.02), 0.438	-	1.01 (0.99–1.03), 0.148	-
ET-1	2.02 (0.96–4.25), 0.061	25.1 (1.03–611), 0.047	1.31 (0.57–2.94), 0.525	-
Gal-3 > 11.5 ng/mL	1.06 (0.96–1.17), 0.266	-	7.11 (2.11–24.0), 0.002	1.12 (1.01–1.25), 0.033
GDF-15	1.74 (0.69–4.37), 0.237	-	0.75 (0.32–1.74), 0.508	-
hsTnT	1.97 (1.06–3.66), <0.001	1.12 (1.03–1.20), 0.010	1.01 (0.96–1.05), 0.959	-
IL-6R ά	1.03 (1.01–1.05), 0.009	1.07 (1.02–1.10), 0.006	1.01 (0.98–1.03), 0.481	-
PCSK9	1.00 (0.99–1.01), 0.474	-	1.03 (0.99–1.08), 0.129	-
NT-pro-BNP > 500 ng/mL	5.21 (1.49–18.1), 0.009	1.54 (0.24–10.1), 0.647	1.00 (0.99–1.04), 0.345	-
uPAR	1.05 90.87–1.27), 0.581	-	0.99 (0.74–1.33), 0.987	-
Serum creatinine	1.02 (1.01–1.04), 0.009	1.06 (1.0–1.13), 0.039	1.01 (0.98–1.03), 0.326	-
eGFR	0.97 (0.95–0.99, 0.037	0.87 (0.78–1.00), 0.015	0.97 (0.95–1.04), 0.053	0.98 (0.96–1.01), 0.377
LDL	0.83 (0.49–1.41), 0.496	-	1.26 (0.85–1.87), 0.247	-
HDL	0.47 (0.11–2.09), 0.322	-	0.96 (0.29–3.07), 0.946	-
hs-CRP	0.86 (0.60–1.22), 0.407	-	1.00 (0.77–1.15), 0.947	-

CI, Confidence interval; HR, Hazard Ratio.

## Data Availability

The data presented in this study are available on request from the corresponding author. The data are not publicly available due to privacy.
